# For small (1-3cm) nonfunctional adrenal incidentaloma (NFAI), which option is more appropriate for conservative treatment or surgery?

**DOI:** 10.3389/fendo.2023.1119251

**Published:** 2023-02-01

**Authors:** Xuwen Li, Song Xiao, Xiangpeng Zhan, Yue Yu, Cheng Zhang, Haibo Xi, Gongxian Wang, Xiaochen Zhou

**Affiliations:** Department of Urology, The First Affiliated Hospital of Nanchang University, Nanchang, China

**Keywords:** nonfunctional adrenal incidentaloma, conservative treatment, surgery, efficacy, safety

## Abstract

**Objective:**

To compare the efficacy and safety between conservative treatment and surgery for the patients with small (1-3cm) nonfunctional adrenal incidentaloma (NFAI).

**Methods:**

The patients with small (1-3cm) NFAI who received conservative treatment or surgery in our hospital from November 2018 to December 2019 were retrospectively collected. A total of 83 patients were included in this study. They were divided into two groups according to the treatment methods: the surgery group (n=51) and the conservative treatment group (n=32).Then patients’ demographics, tumor characteristics, functional indicators and complications were compared. Statistical analysis was performed using t-test for continuous variables and Pearson chi-square test or Fisher’s exact test for categorical variables.

**Results:**

At the time of diagnosis, after 3 months, after 6 months, after 12 months, and after 24 months, we found that there was no significant difference between the two groups in systolic blood pressure, diastolic blood pressure, serum potassium levels, and hormone levels. 51 patients chose to have surgery, of which 41 patients chose RLA and 10 patients chose RARLA. RARLA group patients had the highest total cost and conservative treatment group patients had the lowest cost, and the difference was significant (P < 0.001). There was no significant difference in tumor size in the conservative treatment group between at the time of diagnosis and after 24 months (P = 0.305).

**Conclusion:**

Surgical treatment is more effective for 1-3cm NFAI, but conservative treatment is safer and more economical. Follow-up after conservative or surgical treatment is necessary.

## 1 Introduction

Adrenal incidentaloma (AI) refers to the adrenal tumor whose diameter ≥ 1cm is accidentally found by imaging examination during physical examination or diagnosis and treatment of other non-adrenal diseases (1, 2). AI is a common disease, accounting for 8.7% of the autopsy population (3). And as people aged, the incidence in the elderly population increased to 10% (4). Approximately 80% of AI are benign and have no endocrine function, while 20% are malignant and have endocrine function (5). Nonfunctional adrenal incidentaloma (NFAI) is adrenal tumor that has no endocrine function at the time of diagnosis, accounting for roughly 75% of all AI (5). Almost all patients with NFAI have no symptoms, but one study found a 41% long-term incidence of hypertension in patients with NFAI (6). Due to the fact that NFAI is mostly benign, and considering the trauma and risk of surgery, a basic consensus in the past has been to recommend conservative treatment for tumors between 1 and 3cm with no endocrine function (1, 7). However, with the development of minimally invasive concept and the progress of surgical technology and equipment, the trauma and risk of surgery gradually decreased (8, 9). Doctors and patients are becoming increasingly concerned about the tumor’s malignant potential, the potential for hormone secretion, and the potential close relationship with hypertension. Patients with small adrenal tumors discovered in an outpatient clinic are increasingly requesting or being recommended surgical treatment. However, for these small NFAI, conservative treatment or surgical treatment is still being discussed by endocrinologists and urologists.

In order to compare the efficacy and safety of conservative treatment and surgical treatment for these patients with small NFAI, we carried out this study. We hope to provide new data for the treatment of these patients through this study.

## 2 Materials and methods

### 2.1 Data source and ethics statement

Our prospectively maintained database was retrospectively scrutinized to acquire details regarding the baseline demographic and clinical information after obtaining the approval of the institutional review board and ethics committee of the First Affiliated Hospital of Nanchang University.

### 2.2 Patient selection

The patients with small (1-3cm) NFAI who received conservative treatment or surgery in our hospital from November 2018 to December 2019 were retrospectively collected. The patients were included in this analysis according to the following inclusion criteria: [1] Patients diagnosed as adrenal tumor; [2] The tumor size was 1-3 cm; [3] Unilateral tumor; [4] At the initial diagnosis, the serum potassium was between 3.5-5.5 mmol/L; [5] the levels of aldosterone, cortisol and VMA were within the normal range. The exclusion criteria were as follows: [1] During hospitalization, other operations other than adrenalectomy were performed; [2] systolic blood pressure exceeded 140 mmHg at initial diagnosis; [3] diastolic blood pressure exceeded 90 mmHg at initial diagnosis; [4] accompanied by other serious complications.

### 2.3 Technical considerations

Patients in the conservative treatment group did not receive any medical or lifestyle interventions. All the patients in the surgery group were operated by the same medical team, and the surgical methods were divided into retroperitoneal laparoscopic adrenalectomy (RLA) and robot assisted laparoscopic adrenalectomy (RARLA) according to the characteristics of the tumor. All patients underwent follow-up examinations at 3, 6, 12 and 24 months after diagnosis or surgery. All patients were asked to sign an informed consent form.

### 2.4 Variables and endpoints

Variables in the study include demographic characteristics (age, sex, body mass index (BMI)), serum potassium level, blood pressure level, hormone level (aldosterone, cortisol, Urine Vanilla Amygdalic Acid (VMA)), tumor characteristics (tumor size, tumor site), treatment methods (surgical treatment, waiting for observation), and other variables (total cost).

The primary end points of this study were to compare the average differences in tumor size, blood pressure and hormone levels between the surgery group and the conservative treatment group at the time of diagnosis, after 3 months, after 6 months, after 12 months, and after 24 months. The secondary endpoint was the total cost of the two groups, which was defined as the cost of hospitalization and outpatient services.

### 2.5 Statistical analysis

Means and standard deviations were determined for the normally distributed continuous variables, while those with nonnormal distribution were presented as median and interquartile range. Categorical variables were presented as frequencies and their proportions. The Student’s t-test was performed for the normally distributed continuous variables. The Mann-Whitney test was employed to analyze VMA between the surgery group and the conservative treatment group. Analysis of variance was used to compare the total cost among conservative treatment, Retroperitoneal laparoscopic adrenalectomy (RLA) and Robot assisted laparoscopic adrenalectomy (RARLA) groups. All categorical variables were compared with the Chi square test. SPSS 26.0 (IBM Corp, Armonk, NY) was utilized for all statistical analysis with a two-sided p value < 0.05 denoting statistical significance.

## 3 Results

According to the inclusion and exclusion criteria, 81 patients were included in this study from November 2018 to December 2019. Thirty-two patients with unilateral nonfunctioning adrenal incidentaloma chose conservative treatment, while 51 patients chose to undergo surgery, of which 41 patients chose RLA, and 10 patients chose RARLA. There was no significant difference in baseline characteristics between the two groups (**Table 1**).

**Table 1:** Baseline demographical and clinicopathological characteristics of patients.

Variables	Adrenalectomy(n=51)	Watchful Waiting(n=32)	*P*
Age, year, mean (SD)	49.3(10.6)	49.6(11.1)	0.912
Gender, n (%)			0.790
Male	19(37.3%)	11(34.4%)	
Female	32(62.7%)	21(65.6%)	
BMI, kg/m^2^, mean (SD)	23.9(3.1)	24.8(3.1)	0.221
Tumor size, cm, mean (SD)	2.1(0.5)	2.0(0.5)	0.237
Tumor site, n (%)			0.849
Left	25(49.0%)	15(46.9%)	
Right	26(51.0%)	17 (53.1%)	

SD, standard deviation; BMI, body mass index.

By comparing the average difference between the two groups of patients at the time of diagnosis, after 3 months, after 6 months, after 12 months, and after 24 months, we found that there was no significant difference between the two groups in systolic blood pressure, diastolic blood pressure, serum potassium levels, and hormone levels (**Table 2**). After 24 months, only 2 (3.9%) patients in the surgery group had an increase in aldosterone, while the conservative treatment group was 3 (9.4%). However, 2 (3.9%) patients in the surgery group had increased cortisol, compared with 4 (12.5%) in the conservative treatment group.

**Table 2:** Comparison of follow-up results of adrenalectomy group and watchful waiting group.

Variables	Adrenalectomy(n=51)	Watchful Waiting(n=32)	*P*
Systolic pressure, mmHg, mean (SD)			
At diagnosis	119.63(12.92)	118.53(14.85)	0.723
After 3 months	114.80(10.90)	114.34(9.23)	0.835
After 6 months	118.57(8.50)	117.00(7.50)	0.395
After 12 months	121.10(5.26)	120.88(4.09)	0.839
After 24 months	122.12(7.30)	122.06(9.66)	0.976
Diastolic pressure, mmHg, mean (SD)			
At diagnosis	78.51(10.53)	77.47(12.19)	0.681
After 3 months	72.29(6.06)	72.47(5.35)	0.894
After 6 months	76.90(6.90)	75.94(5.60)	0.508
After 12 months	76.16(4.97)	75.31(4.93)	0.452
After 24 months	75.39(10.96)	77.88(7.69)	0.230
Serum potassium, mmol/L, mean (SD)			
At diagnosis	3.88(0.25)	3.94(0.31)	0.301
After 3 months	3.81(0.25)	3.77(0.21)	0.509
After 6 months	3.94(0.34)	3.94(0.41)	0.978
After 12 months	4.21(0.28)	4.16(0.24)	0.462
After 24 months	4.36(0.37)	4.22(0.48)	0.160
Increased aldosterone, n (%)			
At diagnosis	0	0	–
After 3 months	0	0	–
After 6 months	0	1(3.1%)	0.386
After 12 months	0	3(9.4%)	0.105
After 24 months	2(3.9%)	3(9.4%)	0.588
Increased cortisol, n (%)			
At diagnosis	0	0	–
After 3 months	0	0	–
After 6 months	0	2(6.3%)	0.146
After 12 months	2(3.9%)	3(9.4%)	0.588
After 24 months	2(3.9%)	4(12.5%)	0.301
VMA, mg, median (25th–75th percentile)			
At diagnosis	9.04(6.83-12.82)	7.37(3.78-11.11)	0.064
After 3 months	8.90(6.52-11.39)	7.08(3.90-11.36)	0.086
After 6 months	7.79(6.70-9.62)	6.75(5.97-9.96)	0.091
After 12 months	7.25(5.45-9.47)	6.54(4.72-9.62)	0.405
After 24 months	8.50(4.24-12.21)	9.45(6.60-11.76)	0.446

SD, standard deviation; VMA, Urine Vanilla Amygdalic Acid.

**Figure 1:**
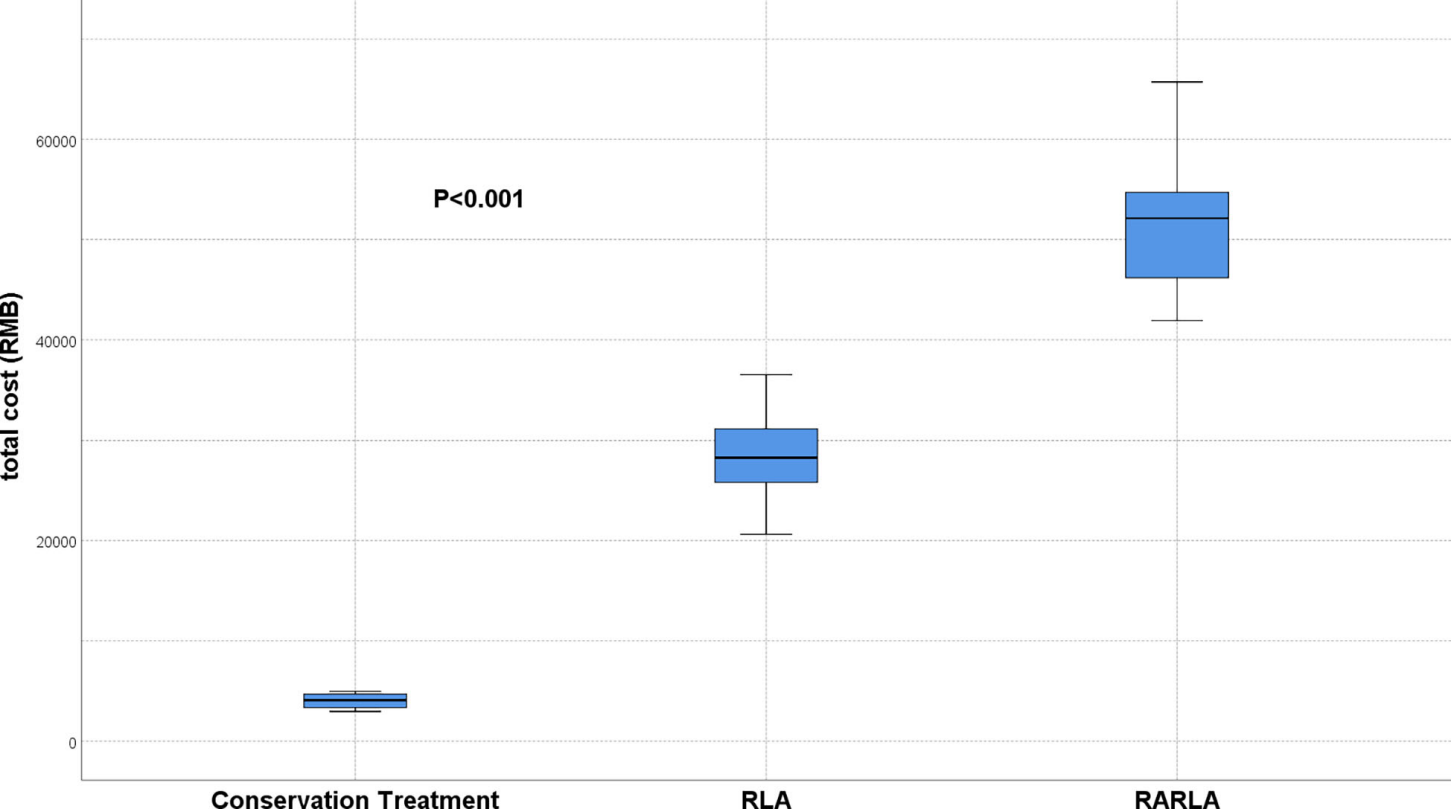
Comparison of total costs in the conservative treatment, LPA and RALPA groups. The total costs of conservative treatment was the least, while RALPA was the most.

**Figure 2:**
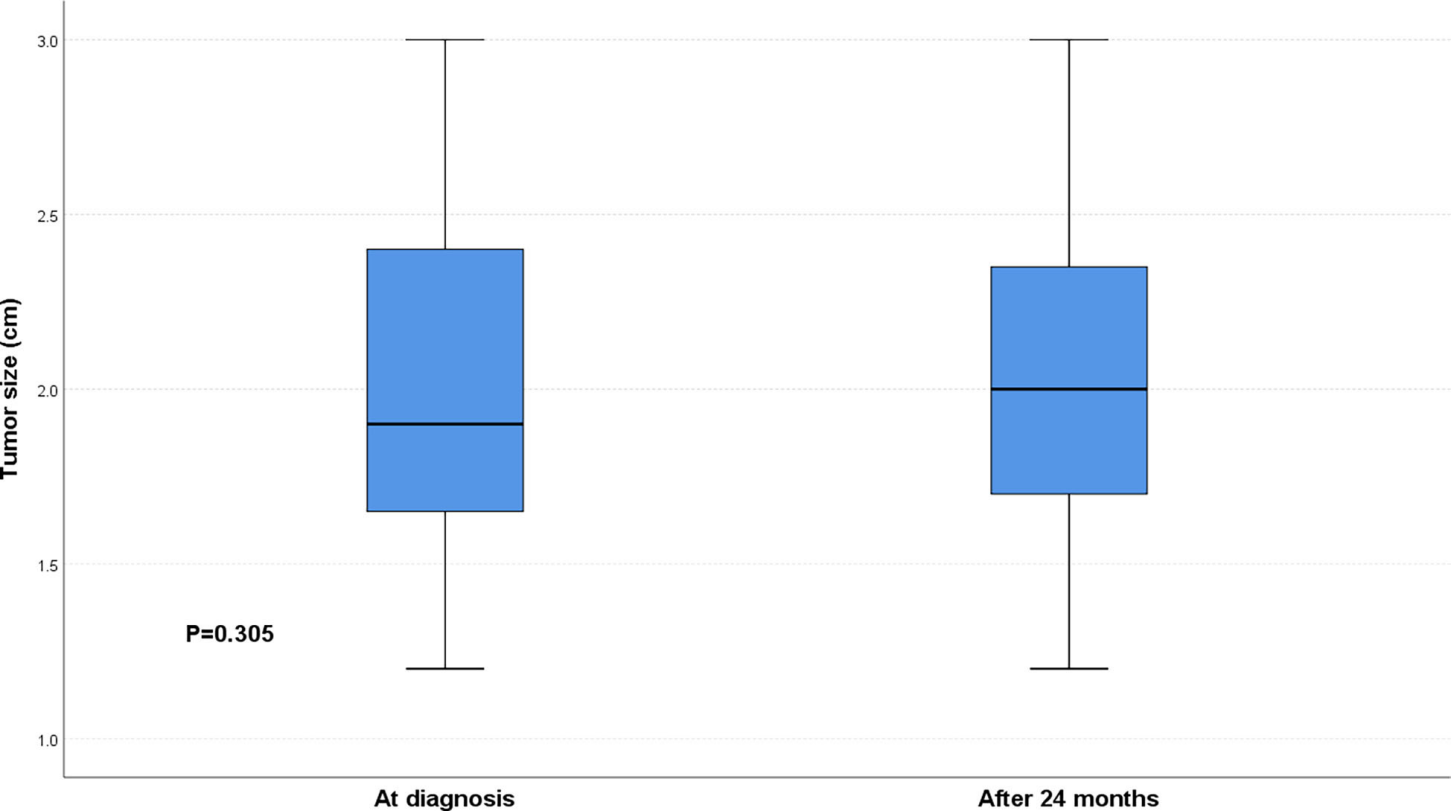
Comparison of tumor size at diagnosis and 24 months later in the conservative treatment group. Within 24 months, patients in the conservative treatment group showed no significant tumor growth.

51 patients chose to have surgery, of which 41 patients chose RLA and 10 patients chose RARLA. We compared the total cost of conservative treatment, RLA and RARLA groups and found that RARLA groups patients had the highest total cost and conservative treatment groups patients had the lowest cost, and the difference was significant (P < 0.001) (**Figure 1**). There was no significant difference in tumor size in the conservative treatment group between at the time of diagnosis and after 24 months (P = 0.305) (**Figure 2**).

## 4 Discussion

Physical examination has become a service item accepted by an increasing number of people as people pay more attention to their health. The growing popularity of physical examinations, as well as the accompanying increase in imaging, is a major reason for the rising incidence of adrenal incidentaloma (AI) year after year (10). Nonfunctional adrenal incidentaloma (NFAI) are adrenal tumors that have no endocrine function at the time of diagnosis, accounting for roughly 75% of all adrenal incidentaloma (5). Although most NFAI are benign and non-functional, with no obvious clinical symptoms, it has become an important disease affecting human health, attracting the long-term attention of endocrinologists and urologists due to its relatively high incidence in the population and the potential for malignant lesions (3, 4, 11). Endocrinologists and urologists share the goal of finding an effective, safe, and cost-effective treatment.

Previously, the general consensus was to surgically resect NFAI larger than 3cm and to treat tumors smaller than 3cm conservatively (7). Of course, as with other types of tumors, size is not the sole determinant of treatment. Another general agreement is that when a NFAI becomes functional or significantly grows in size during conservative treatment, surgical treatment is a better option than conservative treatment (10, 12).

However, as research on NFAI progresses, its potential impact on people’s health becomes clearer. NFAI, on the one hand, are thought to be risk factors for cardiovascular disease. Tuna (13) compared the prevalence of hypertension in the general population to patients with NFAI in one study. The study’s findings revealed that the incidence of hypertension in patients with NFAI was significantly higher than in the general population. Furthermore, Cansu and his colleagues revealed in a paper that NFAI can increase patients’ cardiovascular risk, and that this effect may be related to carotid intima media thickness (CIMT), pulse wave velocity (PWV), augmentation index (AIx) (14). This viewpoint was supported by Emral’s research (15). NFAI, on the other hand, are thought to be closely related to metabolic disorders. Ribeiro Cavalari (16) compared the prevalence of metabolic syndrome in NFAI to that of the general population in one study. The incidence of NFAI was 69.2% versus 31.0% in the general population, indicating a significant difference between the two. In another study, patients with NFAI were found to have a higher risk of insulin resistance and diabetes (17). Furthermore, NFAI can cause a decrease in serum adiponectin levels as well as an (17) increase in blood lipid levels, affecting bone metabolism (18) and the appearance of periodontal disease (19).

According to the above studies, even if the NFAI does not have significant cortisol and aldosterone overproduction, it can still have a negative impact on our health in a variety of ways. This appears to imply that early surgical treatment for patients with NFAI can prevent further damage. Our findings appear to support this viewpoint, with increased aldosterone and cortisol occurring in 3.9% of the surgery group after 2 years, compared to 9.4% and 12.5% in the conservative group, respectively. Although our findings did not indicate hypertension in the conservative treatment group, this could be due to the shorter follow-up period.

Safety and cost are also important considerations in treatment selection. Conservative treatment for small NFAI was previously recommended because it was safer and less expensive than surgery. However, with the introduction of laparoscopic (8) and Da Vinci surgical robots (9), the risk of surgery has decreased significantly. That is one of the reasons why an increasing number of patients are undergoing surgery. Of course, the risk of surgery remains higher than the risk of conservative treatment; after all, surgery is a traumatic procedure. Our findings show that conservative treatment is more cost-effective, whereas patients undergoing robot-assisted abdominal surgery as part of the surgical approach face greater financial strain.

In conclusion, for patients with 1-3cm NFAI, surgical treatment is more appropriate in terms of therapeutic effect. Conservative treatment may be an option if safety or economic factors are taken into account; after all, as our study demonstrated, conservative treatment did not significantly increase the size of the tumor within two years.

## 5 Conclusions

Surgical treatment is more effective for 1-3cm NFAI, but conservative treatment is safer and more economical. Follow-up after conservative or surgical treatment is necessary.

## Data availability statement

The original contributions presented in the study are included in the article/Supplementary Material. Further inquiries can be directed to the corresponding authors.

## Ethics statement

The studies involving human participants were reviewed and approved by the Ethical Committee of The First Affiliated Hospital of Nanchang University. The patients/participants provided their written informed consent to participate in this study.

## Author contributions

Conception and design: GW and XCZ. Surgeons: GW and XCZ. Acquisition of data: SX and XPZ. Preparation of tools: YY and CZ. Analysis and interpretation of data: XL and XPZ. Drafting of the manuscript and statistical analysis: XL and SX. Critical revision: GW and HX. Obtaining funding: XCZ and HX. All authors contributed to the article and approved the submitted version.
